# Quantitative Measurement of Plasma Free Metanephrines by a Simple and Cost-Effective Microextraction Packed Sorbent with Porous Graphitic Carbon and Liquid Chromatography-Tandem Mass Spectrometry

**DOI:** 10.1155/2021/8821276

**Published:** 2021-02-04

**Authors:** Xin Xiong, Yuanyuan Zhang, Rongsheng Zhao

**Affiliations:** ^1^Department of Pharmacy, Peking University Third Hospital, Beijing 100191, China; ^2^Therapeutic Drug Monitoring and Clinical Toxicology Center of Peking University, Beijing 100191, China

## Abstract

Plasma free metanephrines are widely used for the diagnosis of pheochromocytoma and paraganglioma (PPGL), yet quantifying metanephrines using a simple and cost-effective approach may be challenging due to preanalytical and analytical constraints. In this study, we established and validated a new method for quantitative measurement of plasma free metanephrines based on microextraction by packed sorbent (MEPS) with porous graphitic carbon (PGC) and liquid chromatography-tandem mass spectrometry (HILIC-MS/MS). The elution step was fully compatible with HILIC mode without evaporation and reconstitution. The analytes were well resolved, and potential interferences (54 substances) were investigated. This method was linear from 24.7–2717 pg/mL for metanephrine (MN) and 24.5–4010 pg/mL for normetanephrine (NMN) with a coefficient of determination (*R*^2^) higher than 0.994. The limit of MN and NMN detection were 12.4 pg/mL and 12.3 pg/mL, respectively. The intra- and interassay impressions were ≤12.8% for spiked quality controls and ≤13.6% for commercial quality controls; the method recoveries ranged within 88.0–109.0%, respectively. The area under the receiver operating characteristic (ROC) curve was 0.848 ± 0.047 for MN and 0.979 ± 0.021 for NMN. Validation that was performed by comparing clinical specimens with various biochemical results showed that plasma free metanephrines in a seated position had comparable sensitivity and lower specificity to urinary free metanephrines, which could be compensated by combining other biochemical tests. The newly developed MEPS method resulted as a time-saving, reliable, and cost-effective microextraction technique that can be applied for a successful screening of PPGL.

## 1. Introduction

Pheochromocytoma and paraganglioma (PPGL) are rare neuroendocrine tumors characterized by the overproduction of catecholamines [1], which can lead to serious cardiovascular complications, such as hypertensive crisis, myocardial infarction, and stroke [2]. The biochemical testing is crucial for diagnosing PPGL. Compared with plasma catecholamines and urinary catecholamines, plasma free metanephrines (O-methylated metabolites of catecholamines) provide higher sensitivity and specificity for the diagnosis of these tumors and thus have been recommended as first-line testing along with urinary fractionated metanephrines [3]. These measurements commonly include metanephrine (MN) and normetanephrine (NMN), the O-methylated metabolite of epinephrine (EPI), and norepinephrine (NE).

Liquid chromatography with electrochemical detection (LC-ECD) is one of the commonly used methods for measuring plasma metanephrines [4, 5], yet this approach requires large sample volumes, laborious sample preparation, and long chromatographic time to achieve the sensitivity. Immunoassay-based methods provide an alternative to LC-ECD; however, the nonspecific binding and cross-reactivity cannot be avoided using this approach [6, 7]. Over recent years, liquid chromatography with tandem mass spectrometry (LC-MS/MS) has been widely used to measure plasma free metanephrines due to its inherent selectivity and sensitivity [8–16]. However, these compounds are poorly ionizable and with high polarity, which presents at very low concentrations in a complex matrix. Hence, an effective, simple, sensitive, and cost-effective sample cleanup for the complex matrix prior to the LC-MS/MS analysis is crucial.

Over the past decades, general solid-phase extraction (SPE) has been often used as the sample preparation step in the analysis of plasma metanephrines [9–16]. Weak cation-exchange SPE sorbents (WCX) have become popular for the isolation of metanephrines from biological samples [10–16]. Besides, the ion-pairing reagent can also be used to form a complex with metanephrines, which could be retained on the C18 SPE cartridges [9]. In these studies, extract evaporation for concentration or use nonvolatile salts as buffers or a large volume of sample (500 *μ*L [9] or 900 *μ*L [12]) and injection volume (35 *μ*L [11] or 40 *μ*L [9]) were required to achieve the necessary sensitivity and specificity. These procedures can increase the complexity and time requirements and shorten the life of the analytical column.

Porous graphitic carbon (PGC), which has particular physical and chemical properties, was developed as a stationary chromatographic phase for LC in the early 1980s. The very polar compounds, including ionized molecules, can be retained on PGC without using ion-pairing agents. This unique property comes from its delocalized band of electrons, enabling stabilizing electronic interactions and direct *π* overlap with diverse analytes [17]. The PGC stationary phases have been exploited to analyze polar pharmaceutical compounds [18], impurities [19], or endogenous metabolites. Currently, the PGC sorbent has become commercially available in SPE and microextraction by packed sorbent (MEPS) formats. MEPS is a miniaturized form of the SPE technique that has been accepted as an attractive option and a powerful sample preparation approach suitable for accomplishing bioanalytical challenges since it emerged in 2004 [20]. The greatest advantage of this method is its clean and straightforward MEPS procedure with a short runtime. It can handle a small sample volume while maintaining sufficient selectivity, precision, and accuracy [21]. However, to the best of our knowledge, only several MEPS methods using the amino-propyl silane (APS) or C18 sorbents have been introduced for the sample cleanup of several biogenic amines [22–24].

The aim of this work was to develop and validate an analytical method for the quantitation of metanephrines by employing simple and specific MEPS of minimal plasma sample compatible with hydrophilic interaction chromatography (HILIC) mode that is more suitable for the LC-MS/MS analysis of hydrophilic compounds [25]. A large number of potential interferences of plasma metanephrines were also investigated. Moreover, this study evaluated its usefulness by comparing it with other metabolites, including urinary free catecholamines, metanephrines, and vanillylmandelic acid (VMA) in a routine clinical setting.

## 2. Materials and Methods

### 2.1. Materials and Chemicals

MN and NMN were purchased from Toronto Research Chemicals (Ontario, Canada). MN-d3 and NMN-d3 were obtained from Cambridge Isotope Laboratories, Inc. (MA, USA). Endocrine plasma controls and plasma calibration standard metanephrines were acquired from Chromsystems (Grafelfing, Munich, Germany). HPLC- (high-performance liquid chromatography-) grade acetonitrile, methanol, formic acid, acetic acid, and ammonium formate were obtained from Thermo Fisher Scientific Inc. (MA, USA). Ultrapure water was obtained using a Milli-Q system (Waters Millipore, MA, USA).

### 2.2. LC-MS/MS Conditions

The LC-MS/MS system was composed of an API 4000+ triple quadrupole mass spectrometer (AB Sciex Instruments, CA, USA) equipped with a Shimadzu LC-30A HPLC system (Kyoto, Japan). The mass spectrometer was operated in the positive electrospray ionization (ESI) mode with multiple reaction monitoring (MRM). The column oven was kept at 40°C, and the autosampler was set at 4°C. The chromatographic separation was performed on a ZIC-HILIC column (150 mm × 2.1 mm, 3.5 *μ*m) from Merck (Darmstadt, Germany). The analytes were eluted by gradient mobile phases containing acetonitrile (Mobile Phase A) and 40 mM ammonium formate (Mobile Phase B, pH 3.0). The initial LC gradient of 83% A was linearly decreased to 75% within 3 min and held for 1 min. The gradient was then quickly ramped to 60% A in 0.1 min and held for 1 min; finally, the column was equilibrated at 83% for 3 min. The flow rate was 0.35 mL/min, and the cycle time was 8 min. The injection volume was 5 *μ*L.

Analyst software, version 1.6.2, from AB Sciex was used for instrument control and analysis. The ion spray voltage was optimized to 1500 V at 550°C, and the curtain gas was maintained at 45 psi. The nebulizer gas and the heater gas were set at 40 psi, while the collisionally activated dissociation (CAD) gas was kept at 12 psi. The MRM transitions and compound parameters are summarized in Table S1, with 100 ms dwell time for each transition.

### 2.3. Standards and Specimens

MN and NMN standard stock solutions were prepared in 0.2 M acetic acid at a concentration of 1 mg/mL. Optimization of ion source parameters was performed by infusing a metanephrines solution (500 ng/mL in 50% methanol containing 0.2 M acetic acid) at a flow rate of 5 *μ*L/min. The internal standard (IS) working solution, including MN-d3 (5 ng/mL) and NMN-d3 (40 ng/mL), was prepared in 0.2 M acetic acid in water.

The Chromsystems plasma calibration standards were reconstituted according to the reagent instruction. A six-calibrator curve was prepared and stored at −20°C until use. This material was stable for at most 3 months. For the method validation, quality control (QC) samples were prepared at three concentrations (low: 100 pg/mL for MN, 150 pg/mL for NMN; middle: 500 pg/mL for MN, 750 pg/mL for NMN; high: 2000 pg/mL for MN, 3500 pg/mL for NMN) of plasma samples. For the routine clinical analysis, normal-concentration (60 pg/mL for MN and 100 pg/mL for NMN) and pathological-concentration (1500 pg/mL for MN and 7000 pg/mL for NMN) controls were also prepared by reconstituting Chromsystems normal- and pathological-range controls according to the reagent instruction, respectively. Subsequently, pathological-range control was diluted 1 : 2 with blank plasma from Chromsystems to ensure that NMN fell within the linearity range. Both controls were aliquoted and kept at −20°C for storage up to 2 months.

### 2.4. Sample Collection and Preparation

EDTA plasma specimens were selected from samples submitted for clinical testing. Samples were de-identified and managed according to the guidelines approved by the Institutional Review Board of the Peking University Third Hospital.

A type of MEPS containing PGC sorbents (particle size: 30 *μ*m; pore size: 250 Å; Thermo Fisher Scientific, Australia) and a hand-held automated analytical syringe (eVol; Thermo Fisher Scientific, Australia) was employed for the plasma extraction. The new sorbent was activated in 3 × 200 *μ*L of methanol and 3 × 200 *μ*L of water before extraction. The first step was protein precipitation with methanol. Briefly, 100 *μ*L of methanol was added to 100 *μ*L of plasma sample containing 10 *μ*L of IS working solution. The precipitated sample was then centrifuged at 15000 rpm for 5 min. The supernatant was diluted with 1000 *μ*L of water, and then it was drawn up and down through syringe (7 × 200 *μ*L) without discarding it. The sorbent was washed with 50 *μ*L of water, and analytes were eluted with 40 *μ*L of mobile phase solution. After each extraction, the sorbent was rinsed with 4 × 100 *μ*L of a mixture of acetonitrile:water (8 : 2, v/v, containing 2% formic acid) followed by 2 × 100 *μ*L of water. The MEPS could reuse the sorbents up to 50 times. The schematic diagram of the operation course of MEPS is shown in Figure S1.

### 2.5. Method Validation

The method was validated according to the Clinical and Laboratory Standards Institute (CLSI) C62-A and FDA guidelines. Linearity was assessed by analyzing calibrators at six concentrations on three different days. The lower limit of quantification (LLOQ) was defined as the lowest concentration within ±20% of imprecision and accuracy. The limit of detection (LOD) was defined as the signal-to-noise ratio >3. To evaluate imprecision and accuracy in the plasma analysis, five replicates of three control materials were repeated on three consecutive days (a total of 15 measurements at each level of control). Additionally, normal and pathological levels of QC samples from Chromsystems were processed alongside, and the results were compared with the expected range provided by the manufacturer. Duplicates of each control were assayed for 20 days. We prepared two spiked samples using pooled plasma to investigate the MEPS recovery: prespiked and postspiked. The prespiked sample was prepared by spiking analytes into pooled plasma before MEPS, whereas the postspiked sample was prepared by spiking analytes at the same concentrations as the extract obtained after MEPS of pooled plasma. The percent extraction recovery was calculated as the percent response ratio of each analyte in the prespiked sample to those in the postspiked sample. A postcolumn infusion study was performed to evaluate the matrix effect by injecting the plasma matrix after MEPS (no IS added) onto the LC column and simultaneously injecting the IS standard solution at 10 *μ*L/min into the column effluent. To evaluate the carryover, a set of high (*H*) and low (*L*) samples was assayed in the following consecutive order: 3*L*, 2*H*, 1*L*, 2*H*, 4*L*, 2*H*, 1*L*, 2*H*, 1*L*, 2*H*, 1*L*. Analytical specificity was investigated by analyzing a number of endogenous and exogenous compounds. Individual aliquots of middle QC plasma samples were spiked with solutions of potential interferences. Recommendations from CLSI EP7-A2, Interference Testing in Clinical Chemistry, were used to guide prepared interference concentrations. Plasma sample stability was assessed after 24 h storage at room temperature or in a refrigerator (4°C) for 5 days. The postextracts stored at 4°C in the autosampler for 120 h were also tested. At each storage condition, the determination of samples was repeated in triplicates.

## 3. Results and Discussion

### 3.1. Column Chromatography and MS Condition Optimization

**Figure 1:**
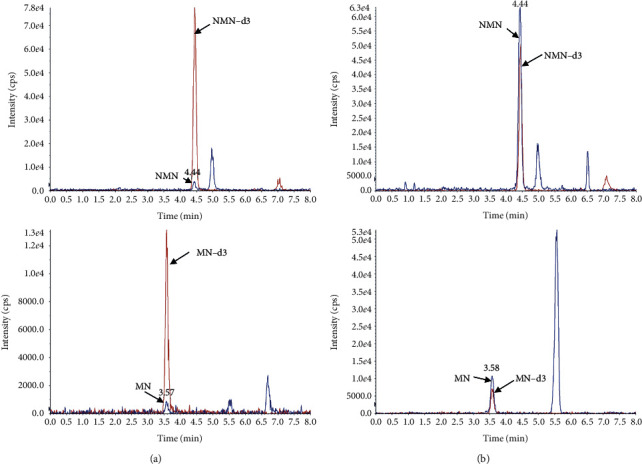
Typical MRM chromatograms of plasma free NMN and MN obtained in a patient without PPGL (a) and a patient with histologically confirmed PPGL (b).

Poor retention on the traditional C18 columns due to high polarity represents one of the challenges when performing chromatographic separation of metanephrines. Another difficulty is well-resolved chromatography of metanephrines and compounds that are structurally similar to them. Although HILIC columns are specifically suitable to retain metanephrines (mixed mode of adsorption and better ionization efficiency) [26], analytical interferences with the measurement of plasma metanephrines in HILIC mode have been recently reported. For example, cross-contamination may occur due to epinephrine/NMN isobaric analyte pairing with identical MRM transitions, 4-hydroxy-3-methoxymethamphetamine (HMMA), a metabolite of 3, 4-methylenedioxymethamphetamine (MDMA), and NMN share a common pharmacophore resulting in the same product ion after fragmentation [27]. An endogenous analyte found in all plasma samples, 3-O-methyldopa, could not be eliminated by multistage fragmentation (MRM^3^) [28], which may enable more specific target quantification compared with traditional MRM [29]. Therefore, chromatographic separation remains essential for interferences involving unresolvable mass fragmentation. Hence, a variety of HILIC-type columns were screened, including Atlantis HILIC silica (150 mm × 2.1 mm, 5 *μ*m) and XBridge Amide (50 mm × 2.1 mm, 3.5 *μ*m) from Waters (Milford, MA, USA), Luna Diol (100 mm × 2.1 mm, 5 *μ*m) from Phenomenex (Torrance, CA, USA), Cosmosil HILIC (100 mm × 2.0 mm, 5 *μ*m) from Nacalai tesque (Kyoto, Japan), and ZIC-HILIC (150 mm × 2.1 mm, 3.5 *μ*m). The ZIC-HILIC column was optimal column for retaining and separating metanephrines with low interferences (detailed in the Analytical Specificity section). Typical chromatograms from a patient without PPGL and those with histologically confirmed PPGL are shown in Figure 1. The two analytes were completely separated within 5.0 min with a runtime of 8.0 min.

MS parameters were optimized using a direct flow injection analysis of 500 ng/mL analyte solution in 0.2 M acetic acid in 50% methanol. As previously reported [24, 30], the more stable ion signals of [M-H_2_O + 1]^+^ for MN and NMN for metanephrines were observed compared with the expected mass/charge (*m*/*z*) ratios of [M + H]^+^ in the ESI source, which was used as the precursor ions. MRM transitions for all the analytes and their deuterated analogues, along with mass detector settings, are described in Table S1.

### 3.2. Optimization of Sample Preparation

The choice of a suitable sample preparation technique for the isolation of metanephrines from complex biological matrices was not straightforward. MEPS is a novel miniaturized SPE with several advantages, such as shorter sample preparation time, miniaturization of sample volume, lower consumption of organic solvents, and absence of extract evaporation. In this study, pooled plasma samples were used for the development and IS for quantification due to the analytes*'* endogenous contribution. Several crucial parameters related to MEPS were screened in this assay.

#### 3.2.1. Choice of Sorbent Type

Since plasma proteins cause clogging of MEPS cartridges, protein precipitation (PP) before loading samples on the MEPS cartridges is often necessary. Several types (methanol, acetonitrile, and mixture) of organic agents were tested in this study. Although a large number of different sorbents are available, the choice of MEPS cartridge is very difficult due to the low retention of metanephrines from the PP solution. First, cation exchange and C18 (particle size: 40–60 *μ*m; pore size: 60 Å; Thermo Fisher Scientific, Australia) cartridges of MEPS, mostly used in SPE, were selected for testing. However, none of these enabled sufficient retention of metanephrines on the sorbent even when the dilution of PP supernatant (up to 20×) contained a large amount of organic solvents. The organic solvents in supernatant reduced the retention of metanephrines.

Another suitable sorbent for hydrophilic compounds was PGC, recently introduced in the MEPS format [31]. Methanol was chosen as the suitable PP solvent because only the 5 : 1 (water:precipitated sample) dilution ratio was needed to enable the retention of metanephrines on PGC sorbents, while a larger dilution ratio was necessary for acetonitrile as a deproteinization agent. Therefore, the PGC sorbent was selected for the MEPS extraction without a large loss of analytes in the loading sample step.

#### 3.2.2. Loading Sample

**Figure 2:**
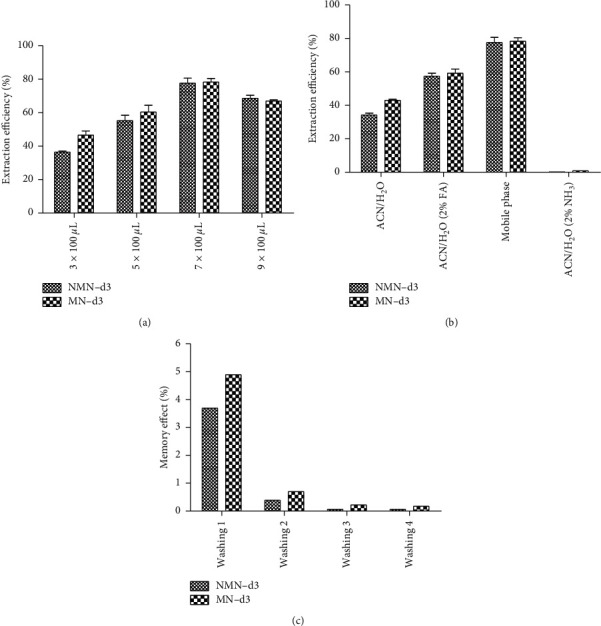
Influence of different parameters on MEPS efficiency: (a) effect of the number of extraction cycles, (b) effect of the elution solution, and (c) carryover of MEPS.

The retention of analytes on the PGC sorbent was affected by the number of extraction cycles. The different number of cycles through which the sample went through efficiently increased the extraction. On the other hand, plasma, a complex matrix, could saturate the sorbent phase and block the access of analytes to the adsorption sites. The result indicated that the seven extraction cycles provided the best conditions for metanephrines retained on the PGC sorbent (Figure 2(a)). An increased number of extraction cycles did not improve the extraction efficiency and might shorten the MEPS sorbent's lifetime.

#### 3.2.3. Washing and Eluting

The sorbent washing step is essential in removing unwanted interferences of the matrices without significant leakage of analyzed compounds. Three different washing solutions (water, 10% methanol in water, and 2% formic acid in water) were assessed for pooled plasma samples. Among these solvents, water was employed as the most appropriate solvent without sacrificing recovery (data not shown). The subsequent stage involved assaying the elution of metanephrines from the sorbent with minimal solvent volume. The elution efficiency was tested using the mobile phase, a mixture solution of acetonitrile and water (8 : 2) containing 2% formic acid, 2% aqueous ammonia, or no additive as solvents (Figure 2(b)). The highest analytical signals among the applied elution solvents corresponded to the mobile phase, which was also the most suitable elution solvent due to the perfect compatibility with HILIC chromatography.

#### 3.2.4. Carryover of MEPS

We finally investigated the carryover of MEPS, which was considered as a decelerating step in sample preparation. The small amount used in the MEPS cartridge can be easily and effectively washed between extractions. A mixture of acetonitrile and water containing 2% formic acid was used as a suitable washing solution (4 × 100 *μ*L) to reduce carryover for reusing of the MEPS sorbent (Figure 2(c)). Carryover was <0.2% prior to reuse.

### 3.3. Method Validation

#### 3.3.1. Linearity and Analytical Sensitivity

The responses of the analytes were linear in the calibration range of 24.7–2717 pg/mL for MN (*R*^2^ > 0.9951) and in the calibration range of 24.5–4010 pg/mL for NMN (*R*^2^ > 0.9942), with a coefficient of variation (CV) of ≤7.3% for the slopes of each analyte on three different days. The F-test for lack-of-fit was also tested for linearity; the results of pure error tests for MN (F^∗^ = 0.887, *P*=0.487) and NMN (F^∗^ = 0.154, *P*=0.959) showed that the relationship was linear for MN and NMN. The LLOQ were 24.7 pg/mL for MN with a CV ≤13.0% and the relative error (RE) ≤−3.2% and 24.5 pg/mL for NMN with a CV ≤11.0% and the RE ≤−1.8%. The LOD for MN and NMN were 12.4 pg/mL and 12.3 pg/mL, respectively, by gradually diluting calibrators.

#### 3.3.2. Precision, Accuracy, and Extraction Recovery

The intra- and interassay imprecision for the spiked QC and Chromsystems QC samples were ≤12.8% and ≤13.6%, respectively. The intraday accuracy for the spiked QC samples was within the range, from 94.0% to 108.1%, with a mean of 99.3%, whereas the interday accuracy was 88.6% to 108.0% with a mean of 101.0%. The intraday accuracy for the Chromsystems QC samples was 88.0% to 103.0% with a mean of 95.9%, and the interday accuracy was 97.2% to 109.0% with a mean of 102.5%. The results confirmed the assay was precise and accurate.

Extract recoveries were evaluated by the traditional postextraction method. The spiked plasma samples were extracted and analyzed along with the nonspiked plasma samples. The extraction recoveries of analytes were higher than 77.6%.

#### 3.3.3. Matrix Effect and Carryover

The deuterated IS solutions' signal intensities were monitored during the infusion study and indicated no significant matrix-induced ion suppression effect at the relevant retention time for all of the tested sample types (Figure S2). The mean (SD) of low MN concentration samples following 60 pg/mL samples was 57.5 (5.1) pg/mL, and the mean (SD) of low concentration samples following 1500 pg/mL samples was 60.7 (7.6) pg/mL, resulting in a carryover of 3.2 pg/mL for MN. The mean (SD) of low NMN concentration samples following 100 pg/mL samples was 104.0 (12.1) pg/mL, and the mean (SD) of low concentration samples following 7000 pg/mL samples was 89.2 (15.9) pg/mL, resulting in carryover of −14.8 pg/mL for NMN. No statistically significant carryover was observed for either analyte.

#### 3.3.4. Analytical Specificity

**Figure 3:**
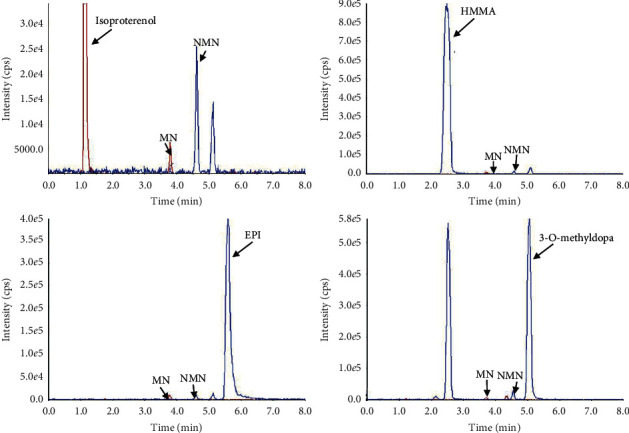
Representative MRM chromatograms of some interferences in human plasma.

Potential interferences, test concentrations, and percent deviation of analyte concentrations in test samples versus respective baseline samples are shown in Table S2 for endogenous and exogenous substances. Petteys *et al.* [11] investigated 28 substances, including structurally related compounds, common drugs, or supplements, and isoproterenol, isoetharine, 3, 4-methylenedioxyamphetamine (MDA), and 3, 4-methylenedioxymethpheamphetamine (MDMA), which may interfere with quantification. In our study, 54 compounds were assessed as potential interferences to ensure the robust performance of the proposed MEPS method, including pseudoephedrine, isoproterenol, isoetharine, labetalol, HMMA, MDA, MDMA, epinephrine, and 3-O-methyldopa interfering with MRM detection of metanephrines [11, 27–29], except for endogenous compounds, vitamins, and frequently used over-the-counter drugs. Levodopa, isoetharine, isoproterenol, and 4-methoxymethamphetamine (PMMA) were identified at retention times of 1.20 min, 2.70 min, 1.13 min, and 1.93 min in the mass transition chromatograms of MN, respectively, but did not interfere with quantification. HMMA, 3-O-methyldopa, and epinephrine were identified at 2.46 min, 5.09 min, and 5.51 min in the mass transition chromatograms of NMN, respectively, but did not interfere with quantification. MDA and a mixture solution of amine were visible at 2.06 min and the range of 1.6-2.2 min in mass transitions of metanephrines, respectively, and did not interfere with quantification. In contrast, these compounds in the mixture could not be identified due to the lack of individual standard reference. Representative chromatograms of some interferences in human plasma are shown in Figure 3. Additionally, labetalol and pseudoephedrine were not visible in this assay because of the selectivity of PGC sorbent. None of the 54 tested compounds interfered with MN and NMN. Below ±15% difference in the concentrations for the QC samples indicated that the quantitation was not affected by the variety of substances.

#### 3.3.5. Stability

Aliquots of plasma samples were analyzed in triplicates stored at room temperature for 24 h, and 4°C for 5 days. The results indicated that *a* ≤14.7% change in all the analytes was obtained under these conditions with satisfactory stability according to the acceptance criterion of ±15% change. Additionally, *a* ≤4.5% change was observed for the postextract at 4°C in the autosampler for 120 h, allowing reanalysis in routine without reperforming MEPS. (Table 1)

### 3.4. Comparison of Analytical Performance by Available Methods

A comparison of the analytical performance of the developed method with published assays is summarized in Table 2. The table shows that the proposed method offers several advantages over previously published methods. First, MEPS was more cost-effective than conventional SPE (MEPS could be reused more than 50 times for plasma samples compared with conventional SPE column or microplate SPE, which could only be used once). Second, MEPS was more ecofriendly compared with the reported sample cleanup. Only relatively small amounts of solvents were needed in MEPS for the washing (<1 mL) and elution (10–50 *μ*L) of the analytes from the sorbent due to ∼2 mg of the solid-packing sorbent. Third, MEPS requires a reduced total analysis time. The novel PGC MEPS sorbent's choice enables using the mobile phase as the elution solvent compatible with HILIC mode. The amount of sorbent was small; sorbent conditioning, evaporation, and reconstitution were unnecessary in MEPS as they were in SPE.

### 3.5. Plasma Metanephrines in Clinical Specimens

**Figure 4:**
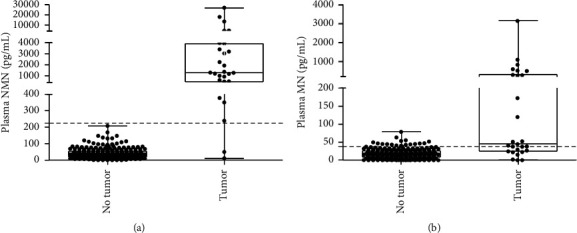
Dot, box, and whisker plots illustrating the concentrations of plasma free metanephrines in clinical specimens. Dotted lines represent the upper cutoff values.

**Figure 5:**
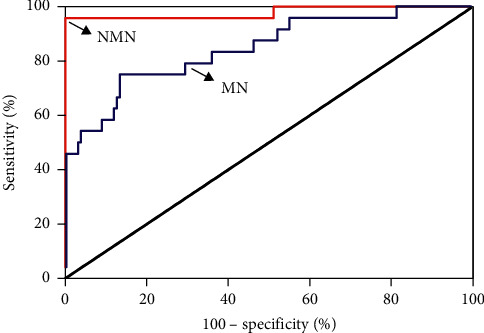
ROC curve for plasma MN and NMN.

In this study, the levels of plasma metanephrines from 161 suspected patients in a seated position were measured using the newly developed method. In 23 patients (7 males; 16 females: 14–64 yrs), the diagnosis of PPGL was based on histological confirmation of surgically resected tumors. Diagnosis of inoperable malignant PPGL in 1 patient (females; age 48 yr) was observed based on the evidence of metastatic disease by imaging studies. Other 137 patients (73 males; 64 females: 14–87 yrs) included primary hypertension, secondary hypertension caused by other factors, and nonfunctioning adrenal tumors diagnosed using clinical or pathological findings that excluded PPGL.  Figure 4 shows the distribution of plasma metanephrines in clinical specimens. Among the 137 patients without PPGL, MN*'*s plasma concentrations ranged from 12.4 to 79.2 pg/mL (median 23.2) and NMN from 12.4 to 207.7 pg/mL (median 42.5). Among 24 patients with PPGL, plasma concentrations of MN ranged from 17.0 to 3161.4 pg/mL (median 52.1) and NMN from 41.6 to 26815.1 pg/mL (median 1273.9). Plasma outputs of NMN among patients with PPGL showed a medium 30-fold increase above medium levels in the control patients and a medium 2.1-fold increase for MN. The area under the receiver operating characteristic (ROC) curve was 0.979 ± 0.021 for NMN and 0.848 ± 0.0047 for MN (Figure 5). Additionally, 95.8% (23/24) had an increase in plasma NMN above the cutoff diagnostic value, compared with 75.0% (18/24) for MN.

Plasma free metanephrines and urinary free metanephrines are recommended for first-line testing of PPGL. Although the plasma test's diagnostic accuracy may be higher than for the urinary test, the difference is relatively small. Yet, no single test can provide an absolute diagnosis of PPGL because a single abnormal metabolite is not a specific index based on the normal concentrations of other metabolites, and the measurement of multiple biochemical analytes can be used for probabilistic elimination for the diagnosis of pheochromocytoma. Therefore, the diagnostic performance of plasma metanephrines was also compared with other biochemical tests in a group of 104 patients, including 21 patients with PPGL, which were determined in our laboratory [26, 32] (Table 3). Urinary free catecholamines and VMA showed a comparable lower sensitivity (71.4%). Combining plasma NMN and MN results demonstrated a comparable diagnostic sensitivity (90.5% versus 90.5%) with urinary free metanephrines but a lower diagnostic specificity (88.0% versus 98.8%). Ideally, samples collected from supine patients compared with seated patients would provide the optimum diagnostic sensitivity and specificity for plasma metanephrines.[3]. However, the inappropriate sampling position is sometimes unavoidable in the clinic, and repeated blood samples tested from a supine position offer a cost-effective approach. The false-positive results could be compensated by combining the tests of plasma metanephrines with urinary metanephrines or catecholamines, or VMA.

## 4. Conclusion

The present study described a simple, miniaturized, and cost-effective analytical method for determining plasma metanephrines using the MEPS sample preparation technique. To the best of our knowledge, a PGC sorbent was used for the first time as the stationary phase for sample preparation of metanephrines. The choice of the novel PGC MEPS sorbent, suitable for the extraction of hydrophilic compounds, enabled us to use the mobile phase as the elution solvent, which was fully compatible with HILIC mode. Therefore, the time needed for the sample processing was significantly reduced without the evaporation and reconstitution steps. The analysis cost was considerably reduced due to the reuse of MEPS cartridges for about 50 extractions, decreased sample volume, solvent, and waste disposal. The reliability and applicability were validated using clinical plasma specimens. This new method may be potentially used as a clinical tool for routine laboratory analysis, where simplicity, rapidity, and small sample volumes are required.

## Figures and Tables

**Table 1:** Intra- and interday assay imprecision and accuracy (spiked QC samples: LQC, MQC, & HQC and Chromsystems QC: C1 & C2).

Compund	Sample	Added/expected (pg/mL)	Intraday					Interday		
			Mean (ng/mL)	SD	CV (%)	Accuracy (%)	Mean (ng/mL)	SD	CV (%)	Accuracy (%)
MN	LQC	100	108	10	9.4	108.0	108	10	9.7	108.0
	MQC	500	481	43	9.0	96.2	516	42	8.2	103.2
	HQC	2000	1899	150	7.9	95.0	2030	152	7.5	101.5
	C1	60	58.9	8.0	13.6	98.2	62.9	8.5	13.4	104.8
	C2	750	708	62	8.7	94.4	729	56	7.7	97.2

NMN	LQC	150	141	18	12.8	94.0	151	16	10.7	100.7
	MQC	750	811	79	9.8	108.1	781	68	8.7	104.1
	HQC	3500	3310	105	3.2	94.6	3100	182	5.9	88.6
	C1	100	103	8	8.0	103.0	109	9	8.6	109.0
	C2	3500	3080	68	2.2	88.0	3460	224	6.5	98.9

**Table 2:** Comparison of the analytical performance of the developed method with five recently described LC-MS/MS methods for determining the concentrations of metanephrines in human plasma.

Sample preparation	Analyte	LLOQ (nmol/L)	Plasma (*μ*L)	Solvent used during extraction (mL)	Potential interferences test	Evaporation	Injection volume (*μ*L)	Reuse number	Ref.
Ion-paring SPE (HyperSep)	NM	0.037	500	5.6	1	Yes	40	1	He et al. (2011)
	NMN	0.098							
SPE (Oasis WCX)	NM	0.020	900	2.7	2	Yes	/	1	Peitzsch et al. (2013)
	NMN	0.024							
SPE (Evolute WCX)	NM	0.070	400	3.0	3	No	30	1	Shen et al. (2019)
	NMN	0.060							
SPE (oasis WCX *μ*Elution)	NM	0.100	200	1.1	28	No	35	1	Petteys et al. (2012)
	NMN	0.100							
SPE (Oasis WCX)	NM	0.104	380	1.1	0	No	20	1	Luo et al. (2017)
	NMN	0.109							
MEPS (PGC)	NM	0.128	100	0.69	54	No	5	50	This work
	NMN	0.134							

**Table 3:** Comparison of biochemical tests for screening for PPGL using clinical specimens.

Clinical specimen metabolite	Plasma metanephrines (pg/mL)			Urinary free catecholamines (*μ*g/day)				Urinary free metanephrines (*μ*g/day)			Urinary VMA (mg/day)
	MN	NMN	MN + NMNM	EPI	NE	DA	All	MN	NMN	MN + NMN	
TP, n	13	19	19	7	13	3	15	10	18	19	15
TN, n	73	83	73	82	82	81	79	82	83	82	78
FP, n	10	0	10	1	1	2	4	1	0	1	5
FN, n	8	2	2	14	8	18	6	11	3	2	6
Sensitivity (%)	61.9	90.5	90.5	33.3	61.9	14.3	71.4	47.6	85.7	90.5	71.4
Specificity (%)	88.0	100	88.0	98.8	98.8	97.6	95.2	98.8	100	98.8	94.0

TP: true positive; TN: true negative; FP: false positive; FN: false negative; EPI: epinephrine; NE: norepinephrine; DA: dopamine.

## Data Availability

The data used to support the findings of this study are included within the article.

## Abbreviations

PPGL: Pheochromocytoma and paraganglioma
MN: Metanephrine
NMN: Normetanephrine
SPE: Solid-phase extraction
MEPS: Microextraction by packed sorbent
LOQ: Limit of quantification
LOD: Limit of detection
MRM: Multiple reaction monitoring
SD: Standard deviation
PGC: Porous graphitized carbon
WCX: Weak cation exchange
APS: Amino-propyl silane
IS: Internal standard
QC: Quality control
ROC: Receiver operating curve.

## Supplementary Materials

Supplementary Materials Table S1: MRM transitions and respective fragmentation parameters for tandem mass spectrometric analysis of metanephrines and their respective deuterated internal standards. Table S2: deviations from baseline concentrations of plasma metanephrines for exogenous and endogenous potential interferences. Figure S1: schematic diagram of operation course of MEPS. Figure S2: postcolumn infusion experiment.
