# Supplementary material for: Quantitative Measurement of Plasma Free Metanephrines by a Simple and Cost-Effective Microextraction Packed Sorbent with Porous Graphitic Carbon and Liquid Chromatography-Tandem Mass Spectrometry
Source: J Anal Methods Chem. 2021 Feb 4;2021:8821276. doi: 10.1155/2021/8821276 (PMC7880714; doi:10.1155/2021/8821276)
Supplement: Supplementary Materials — Table S1: MRM transitions and respective fragmentation parameters for tandem mass spectrometric analysis of metanephrines and their respective deuterated internal standards. Table S2: deviations from baseline concentrations of plasma metanephrines for exogenous and endogenous potential interferences. Figure S1: schematic diagram of operation course of MEPS. Figure S2: postcolumn infusion experiment. [file 8821276.f1.pdf]

Supplementary Materials

Quantitative measurement of plasma free metanephrines by a simple and cost-effective microextraction by packed sorbent with porous graphitic carbon and liquid chromatography-tandem mass spectrometry

Xin Xiong[1,2], Yuanyuan Zhang[1,2], Rongsheng Zhao[1,2*]

[1] Department of Pharmacy, Peking University Third Hospital, Beijing, 100191, China

[2] Therapeutic Drug Monitoring and Clinical Toxicology Center of Peking University, Beijing, 100191, China.

[*] Corresponding author. Fax: +86 10 82266673.
E-mail address: zhaorongsheng@bjmu.edu.cn (R. Zhao)

Table S1. MRM transitions and respective fragmentation parameters for tandem mass spectrometric analysis of metanephrines and their respective deuterated internal standards.

Compound	Q1	Q3	DP (V)	EP (V)	CE (eV)	CXP (V)
MN (quant.)	180	148.1	90	10	23	11
MN (qual.)	180	165.1	90	10	23	11
MN-d3 (quant.)	183	151.1	90	10	25	11
MN-d3 (qual.)	183	168.1	90	10	22	11
NMN (quant.)	166	134.1	60	10	22	11
NMN (qual.)	166	106.1	60	10	32	11
NMN-d3 (quant.)	169	137.1	90	10	23	11
NMN-d3 (qual.)	169	109.1	90	10	30	11

Abbreviations: Q1, precursor ion mass; Q3, product ion masses; DP, declustering potential; EP, entrance potential; CE, collision energy; CXP, cell exit potential.

Table S2. Deviations from baseline concentrations of plasma metanephrines for exogenous and endogenous potential interferences

Exogenous interference and its metabolite (t_R, min)	Test concentration （μg/mL）	MN deviation (%)	NMN deviation (%)	Endogenous interference (t_R, min)	Test concentration （μg/mL）	MN deviation (%)	NMN deviation (%)
Theobromine	100	2.3	-1.3	Tryptophan	200	-12.1	-9.2
Acetaminophen	200	9.8	1.3	Phenylalanine	200	-7.3	0.0
Labetalol	100	10.4	10.7	Tyrosine	1000	3.6	-2.7
Amitriptyline	10	9.0	8.5	Melatonin	165	7.0	-4.7
Pseudoephedrine	10	8.3	2.2	Uric acid	500	-14.6	-10.5
Diphenhydramine	10	-2.5	7.2	Ascorbic acid	200	-10.5	-4.6
Theophylline	100	-1.6	13.5	Urea	2000	-0.9	-5.3
Carbidopa	100	-13.7	-0.5	Glucose	2000	5.8	2.4
Levodopa (**1.20**)	100	11.0	5.7	Riboflavin	108	14.4	7.5
Salicylic acid	1000	0.1	1.3	4-OH-3-methoxyphenyl glycol	1000	1.9	-14.1
Clonidine	10	5.2	13.8	5-Hydroxytryptophol	100	14.5	-4.9
Isoetharine (**2.70**)	100	-7.3	14.4	Nicotinamide	500	-5.2	-13.5
Isoproterenol (**1.13**)	100	8.2	-0.8	Pyridoxal 5-phosphate	500	-5.6	-7.9

3-O-methyl-dopa (**5.09**)	10	-1.6	-0.8	Thiamine	200	4.2	1.2
Methyldopa	100	-9.5	11.9	Nicotinutric acid	200	-13.5	-9.3
MDA (**2.06**)	100	-1.7	5.9	Nicotinic acid	200	-11.3	3.3
HMMA (**2.46**)	100	9.7	-13.7	Pyridoxal	200	-10.7	-9.9
PMMA (**1.93**)	100	-3.6	3.2	5-Hydroxyindoleacetic acid	100	-5.4	2.6
				3-O-methyltyramine	200	-2.4	-4.6
Amine mixtures (phentermine, MDEA, amphetamine, MDMA, MA, MDA) (**1.6-2.2**)	100	5.5	4.4	Norepinephrine	100	9.7	11.1
				3,4-Dihydroxymandelic acid	1000	3.5	13.4
				3,4-Dihydroxyphenylacetic acid	1300	-0.1	3.1
Drug mixtures (Ibuprofen, Naproxen, Cotinine, Nicotine, Caffeine)	1000	13.2	11.3	Epinephrine (**5.51**)	100	-2.7	-12.1
				Dopamine	100	3.5	11.9
				Homovanillic acid	100	6.4	-8.0
				Vanillylmandelic acid	100	14.7	-8.2

MDA: 3,4-methylenedioxyamphetamine; HMMA: 4-hydroxy-3-methoxymethamphetamine; PMMA: 4-methoxymethamphetamine; MDEA: 3,4-methylenedioxyethylamphetamine; MDMA: 3,4-methylenedioxymethpheamphetamine; MA: methamphetamine.

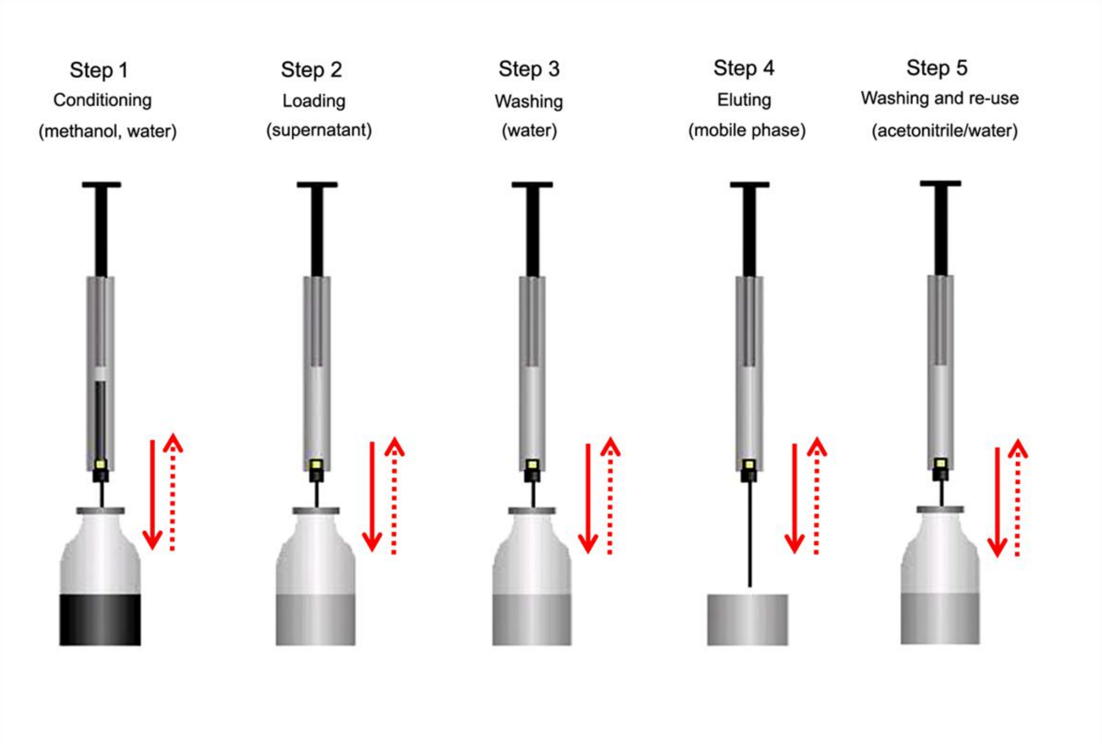

Figure S1 Schematic diagram of operation course of MEPS

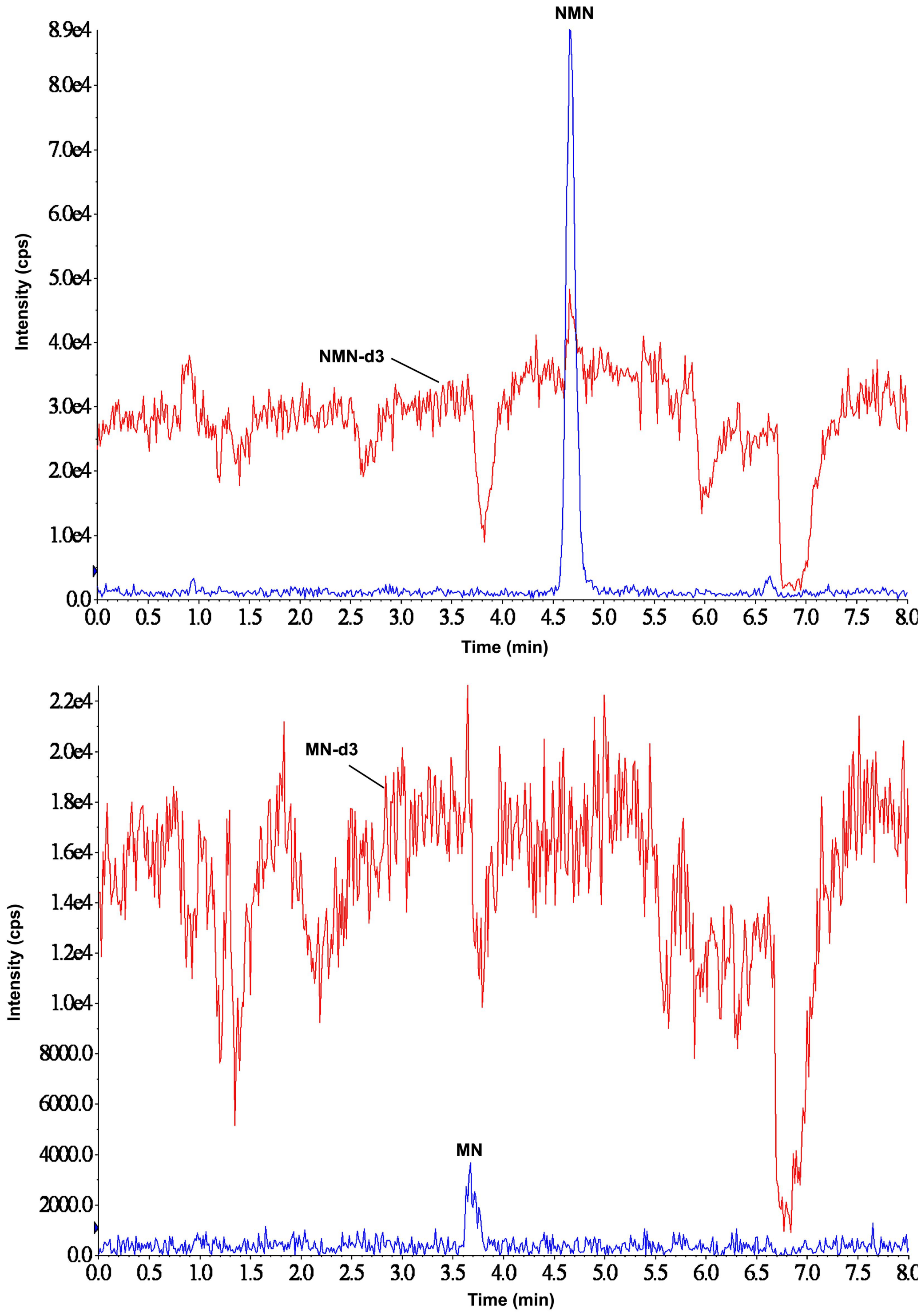

Figure S2 Post-column infusion experiment.
